# Implementation outcomes and associated constructs from the Consolidated Framework for Implementation Research among churches trained online to implement Faith, Activity, and Nutrition in a national implementation study

**DOI:** 10.1093/tbm/ibaf015

**Published:** 2025-05-29

**Authors:** Sara Wilcox, Ruth P Saunders, Andrew T Kaczynski, Caroline Rudisill, Ye Sil Kim, Jasmin Parker-Brown, Kelsey R Day

**Affiliations:** Prevention Research Center, University of South Carolina, Arnold School of Public Health, 921 Assembly Street, Columbia, SC, 29208, USA; Department of Exercise Science, University of South Carolina, Arnold School of Public Health, 921 Assembly Street, Columbia, SC, 29208, USA; Prevention Research Center, University of South Carolina, Arnold School of Public Health, 921 Assembly Street, Columbia, SC, 29208, USA; Prevention Research Center, University of South Carolina, Arnold School of Public Health, 921 Assembly Street, Columbia, SC, 29208, USA; Department of Health Promotion, Education, and Behavior, University of South Carolina, Arnold School of Public Health, 915 Greene Street, Columbia, SC, 29208, USA; Prevention Research Center, University of South Carolina, Arnold School of Public Health, 921 Assembly Street, Columbia, SC, 29208, USA; Department of Health Promotion, Education, and Behavior, University of South Carolina, Arnold School of Public Health, 915 Greene Street, Columbia, SC, 29208, USA; Prevention Research Center, University of South Carolina, Arnold School of Public Health, 921 Assembly Street, Columbia, SC, 29208, USA; Prevention Research Center, University of South Carolina, Arnold School of Public Health, 921 Assembly Street, Columbia, SC, 29208, USA; Department of Exercise Science, University of South Carolina, Arnold School of Public Health, 921 Assembly Street, Columbia, SC, 29208, USA; Department of Public Health Sciences, University of Virginia School of Medicine, 200 Jeanette Lancaster Way, Charlottesville, VA, 22903, USA

**Keywords:** faith-based, churches, organizational change, physical activity, healthy eating, online training

## Abstract

Churches hold promise for reaching populations with high rates of chronic disease, yet few faith-based large-scale implementation studies exist. The study purpose was to examine 12-month implementation outcomes and associated Consolidated Framework for Implementation Research (CFIR) constructs after converting in-person training to online for an evidence-based intervention designed to improve church organizational practices related to physical activity (PA) and healthy eating (HE). US churches recruited from 2020 to 2022 participated in eight online lessons prior to implementation. Each church’s coordinator completed an online baseline and 12-month survey assessing church practices for PA/HE components targeted in the Faith, Activity, and Nutrition (FAN) intervention (opportunities, messages, policies, and pastor support) and constructs from four CFIR domains. Mixed-effects regression models examined changes in practices over time and the impact of in-person versus online church operation at baseline. Linear regression tested associations between CFIR constructs and PA/HE implementation, adjusting for baseline practices. Churches (*N* = 107, 75% predominantly African American) from 23 states enrolled. At 12 months, 84% completed the survey. Implementation of all PA/HE practices increased, with larger effects for churches operating in-person for PA composite, messages, and policies and HE messages and policies. Constructs from all four CFIR domains were associated with implementation outcomes. In conclusion, online training was associated with significantly improved PA/HE church practices at 12 months. For churches operating in-person at baseline, effect sizes and CFIR associations with implementation outcomes were comparable to results of three prior studies using in-person training. Training for FAN is scalable with the potential to advance racial health equity.

## Teaser

Churches trained via online training reported medium to large increases in providing opportunities, policies, messages, and pastor support for physical activity and healthy eating, and factors within the church and individual predicted these increases.

## Graphical Abstract

**Graphical Abstract:**
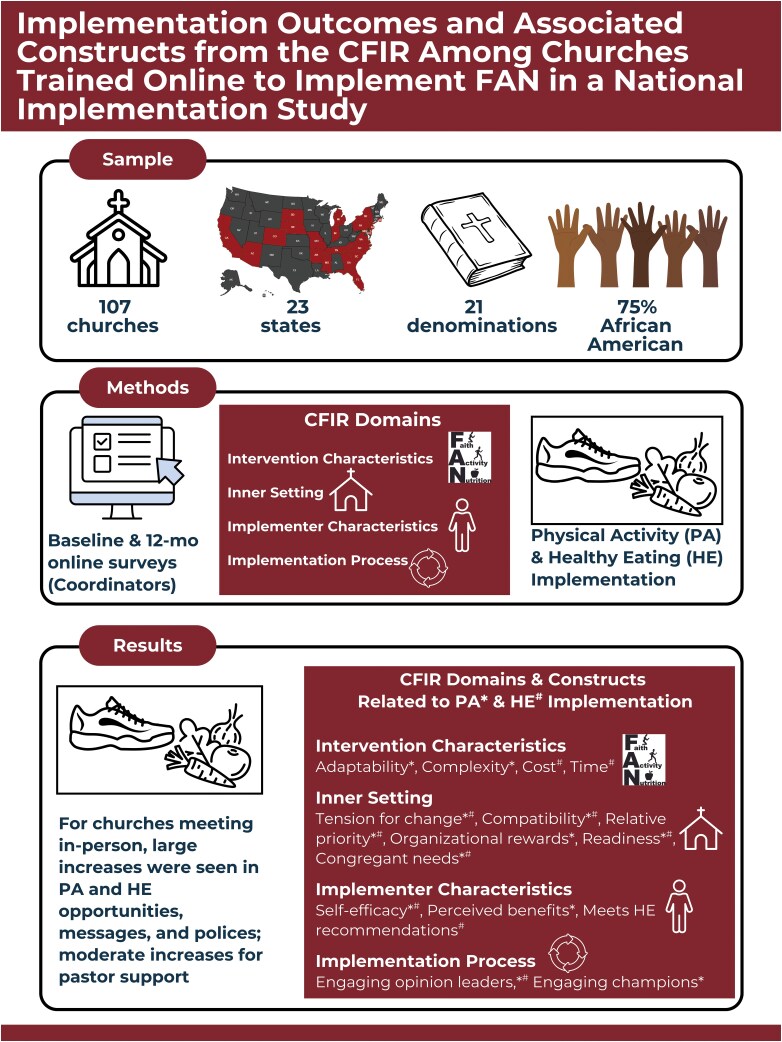

**Implications**

**Practice:** Carefully developed and implemented online training for faith-based interventions improved organizational practices to a similar degree as in-person training.

**Policy:** Effective faith-based programs must consider inner setting factors, factors of the implementation process, and characteristics of implementers to increase implementation of policy, systems, and environmental changes.

**Research**: Further D&I research is needed in faith-based and other community settings, particularly to replicate CFIR and other constructs that serve as facilitators and barriers to implementation outcomes.

## Introduction

Implementation research is critically important to enhance the adoption and implementation of evidence-based interventions in real-world practice, thereby increasing their public health impact [1]. However, most implementation health-related research has been conducted in healthcare settings, with community settings underrepresented despite the community being the place where people spend most of their time [2, 3]. Churches and other faith-based organizations represent a community setting that holds promise for reaching populations that experience higher-than-average rates of chronic conditions, including mid-life and older adults, African Americans, and Southern residents, as these populations also tend to have higher levels of religious participation [4, 5]. Churches, especially African American churches, are natural partners for implementation research given their orientation toward holistic health, the presence of ministries that focus on service and stable lay leadership, their long history of collaborations with public health institutions, and the trust they garner from their members [6, 7]. Further, churches provide a way to reach both urban and rural residents. At the same time, the lack of mandates or incentives for health promotion in this setting, where the primary mission is spiritual development, and the reliance on lay volunteers, pose challenges [3]. While implementation research in faith-based settings is increasing [8–11], it has tended to be relatively narrow in scope (e.g. a small number of churches, one geographic area). Indeed, Parra-Cardona et al. [6] noted that the potential of faith-based organizations to improve health in underserved communities has been underappreciated in implementation science research.

Faith, Activity, and Nutrition (FAN) is an evidence-based intervention that trains and supports committees to change organizational (church) practices related to physical activity (PA) and healthy eating (HE). An effectiveness trial demonstrated that FAN improved organizational practices and increased leisure-time PA and fruit and vegetable intake in South Carolina African Methodist Episcopal congregants [12, 13]. Two subsequent dissemination and implementation studies conducted in South Carolina demonstrated similar positive findings [14, 15]. To scale-up the intervention and make it more widely available, we converted the in-person training to online training and conducted a pilot study [16]. We then conducted a national implementation study of FAN using this online training. Previous papers reported the recruitment strategies and study enrollment and adoption in the national study [17] as well as the response to the online training [18]. The goals of the current paper are to (i) report 12-month implementation outcomes (i.e. fidelity) for the national study; (ii) examine whether implementation outcomes differed according to whether the church was operating only or mainly in-person versus online at baseline (not an a priori study goal, but important as the study began during the COVID-19 pandemic), and (iii) report constructs from the Consolidated Framework for Implementation Research (CFIR) [19] that were associated with implementation outcomes.

## Materials and Methods

### Procedures

This study used a quasi-experimental, single-group design [20]. The University Institutional Review Board reviewed and approved the study protocol and deemed it exempt. Church recruitment strategies, reported in a previous paper [17], were aided by our Community Advisory Board and translation partners, and included flyers, brochures, and a website shared with partners; emails to partners; cold contacts emails to other faith-based networks or health-related organizations with ties to churches; telephone contacts; study presentations; social media and e-media stories and advertisement; paid radio advertising; and word of mouth. Recruitment avenues that would reach predominantly African American congregations were prioritized. A sample size of 100 churches was planned to detect medium effects.

Recruitment materials directed interested churches to an online interest form which, upon completion, generated follow-up from the research staff. Churches were asked to identify a FAN coordinator (i.e. a person to serve as liaison with the research team and coordinate training and implementation efforts in their church) and a church committee to participate in the online training. Eligibility requirements were that the church was in the USA, the pastor provided approval and support to participate, the pastor and FAN coordinator agreed to complete baseline and 12-month online evaluation surveys, and the FAN coordinator agreed to form a church committee for training. Churches were recruited in cohorts, each of which followed the same evaluation and training schedule.

Once a church indicated its intention to enroll in the study, and five weeks before we released the first online training lesson to the cohort, we initiated online baseline surveys. Baseline surveys occurred between August 2020 (Cohort 1) and December 2022 (Cohort 10). Twelve months after churches completed the online training, links to online surveys were emailed. These surveys were completed between January 2022 and March 2024.

### Intervention

The FAN intervention focuses on organizational change within the church setting and was developed using a community-based participatory research process [21]. It is indexed in the National Cancer Institute’s Evidence-Based Cancer Control Program database [22]. The training prepares church committees to implement FAN and covers PA and HE (fruits, vegetables, whole grains, saturated fats, and sodium) basics and recommendations [23, 24] and ideas for how to implement the four PA and HE core components based on Cohen et al.’s structural model of health behavior [25]: (i) increase opportunities, (ii) share messages, (iii) set policies, and (iv) enlist pastor support. In this study, we converted the in-person training to an online format to increase scalability, as described in detail elsewhere [16].

Table 1 describes the primary implementation strategies [2]. We used an online learning management system (Moodle) to house the training. The training site included eight lessons, each with a knowledge check (10 items), feedback survey, and resources; 12 months of materials to support implementation (e.g. newsletters, bulletin inserts, posters, handouts); and an online discussion board [16]. Because recruitment began during the COVID-19 pandemic, we also developed a guide with examples of activities that could still be done even if a church was not meeting in person (e.g. activity breaks in online meetings). More details, including training content, engagement with training, and reactions to the training, are included in another paper [18]. A new lesson was released each week and available if the committee member completed the prior lesson (completion recorded by Moodle), had a satisfactory score on the knowledge check (≥80%; opportunities to retake), and completed the feedback survey.

**Table 1:** Primary implementation strategies used in the national implementation study

Implementation strategy	Description of implementation strategy in FAN
Stage implementation scale-up	The online training was piloted with 9 churches [16] and feedback incorporated before launching the national implementation study.
Centralize technical assistance	Study staff provided technical assistance to churches as needed. Study staff also used the discussion board for churches to request and receive technical assistance, including from other churches.
Adapt and tailor to context—promote adaptability	FAN uses a flexible intervention process. Within the four key components, churches can choose or create activities tailored to their congregation’s unique needs and interests.
Identify and prepare champions	Study staff worked with church leaders to identify a FAN coordinator in their church who is passionate about health.
Organize implementation team meetings	The training encouraged church committees to meet regularly, ideally monthly, to plan for the implementation of FAN.
Use advisory boards and workgroups	Our CAB provided input and advice throughout the study. Our translation partners also offered advice and assistance in sharing the availability of FAN with church and health networks.
Develop and distribute educational materials	We developed an implementation manual, a program plan template, and 12 months of health-related tools and resources.
Make training dynamic	We hired an external group to create visually appealing lessons, with diverse photos, videos, case studies, and interactive elements.
Use train-the-trainer strategies	We trained church committees to work with formal and lay leaders to implement FAN.
Remind implementers	Study staff used the discussion board and emails to remind churches to use monthly resources and submit their program plan.

Implementation strategies are from Balis et al. [2].

Each lesson encouraged committee members to collaborate and draft portions of a program plan that outlined their church’s plans for implementing core intervention components over the next 12 months. The plan was flexible so that churches could choose suggested activities or create activities best suited to their congregation. After training, churches were asked to complete and submit their program plan. Over the next 12 months, the study coordinator moderated the discussion board by posting reminders to utilize the monthly materials, sharing holiday greetings, and replying with encouragement or assistance to committee member posts. Technical assistance was provided by telephone as requested.

### Study measures

#### Church meeting format

Due to the timing of church recruitment and its overlap with the COVID-19 pandemic, questions about how churches were operating changed from the earlier to the later cohorts. However, FAN coordinators were asked consistently across cohorts how they attended church at baseline, with these options: (i) I only go to in-person services and activities, (ii) I sometimes use online services and activities (but mainly go in-person), (iii) I mainly use online services and activities, or (4) I only use online services and activities. We created two groups and used these groups as a proxy for how churches were operating: only/mainly in-person versus only/mainly online.

#### Implementation outcomes (fidelity)

Implementation of the FAN core components, based on Cohen et al.’s model [25], was assessed at baseline and 12 months after completion of training through online surveys with FAN Coordinators. The specific items (11 items each for PA and HE) are shown in Supplementary File 1. FAN coordinators reported the frequency with which their church was implementing each component on a 4-point Likert scale with 1 being the lowest rating (“not at all” or “rarely or never,” depending on the item) and 4 being the highest rating (“almost all of the time” or “about weekly”). This instrument was created in our previous effectiveness trial [12] and used in two previous D&I studies [14, 15], allowing us to make comparisons across studies. We created a mean score to represent the implementation of each of the four components (opportunities, messages, policies, and pastor support). We also created two composite scores, one for PA implementation and a second for HE implementation, that represented an average of the four component mean scores. Finally, within the HE opportunities component score, we also examined whether there were changes in each of the subitems that comprised this component (i.e. changes in providing fruits, vegetables, whole grains, and low-fat food options).

#### Constructs from the CFIR

The CFIR is a widely used implementation science determinants framework that was designed to examine factors associated with implementation outcomes [19, 26]. It is organized into five domains of influence: characteristics of the intervention, outer setting, inner setting, characteristics of implementers, and the implementation process. In a previous study, our research team followed a systematic process to identify constructs most relevant to a church setting, drawing on our extensive history of faith-based research and the broader scientific literature. We identified existing items or, when necessary, created new items that mapped onto these constructs (see [15]). We also identified the optimal time for assessing each construct (baseline, near the end of training, and/or 12 months). We assessed constructs from each domain except the outer setting. In our previous study, we identified two constructs of potential relevance in the faith setting; however, neither was found to relate to implementation and did not seem relevant in this national study. Unless otherwise noted, FAN coordinators rated their agreement with CFIR items on a 4-point Likert scale, ranging from 1 (strongly disagree) to 4 (strongly agree). As needed, items were reverse scored so that a higher score was more favorable and expected to be associated with greater implementation. The domains, constructs, items, and source of items are shown in Supplementary File 2.

From the *intervention characteristics domain*, we assessed adaptability, complexity, cost (both in terms of time and finances), and relative advantage. From the *inner setting domain*, we assessed several structural characteristics: presence of an active health ministry, tenure of the pastor, weekly worship attendance, predominant race of congregation, and whether there was a pastor change during the implementation period. We also assessed culture and networks and communications. Several implementation climate constructs were assessed: tension for change, compatibility, relative priority, and organizational incentives and rewards. Lastly, we assessed the constructs of readiness for implementation (resources and leader engagement) and congregant needs and preferences. From the *characteristics of the individual domain*, we assessed FAN coordinators’ beliefs about the intervention, self-efficacy, perceived benefits, and individual identification with the organization. We also assessed personal attributes of the FAN coordinator: length of church membership, whether they led or co-led health promotion efforts in their church or elsewhere in the past 12 months, age, education, gender identity, and meeting public health recommendations for each target behavior (PA and fruit/vegetable intake). Finally, from the *implementation process domain*, we assessed the constructs of engaging opinion leaders and engaging champions.

### Analyses

#### Implementation outcomes (fidelity)

To assess changes in church practices, a repeated measures analysis was conducted (using SAS PROC MIXED) for the overall PA and HE implementation composite scores and for the components within. Our models included a Time × Meeting format interaction to determine whether changes over time differed by how the church operated at baseline (only/mainly in-person vs. online). We also computed within-group effect sizes to obtain the magnitude of effects over the 12-month period. Cohen’s d was calculated as (12-month least square mean minus the baseline least square mean) / baseline standard deviation. Effect sizes of *d*=|0.20| were considered small, *d*=|0.50| medium, and *d*=|0.80| large [27]. Churches meeting only/mainly online at baseline did not have the opportunity to implement HE opportunities and were therefore excluded from analyses for that component.

#### CFIR constructs associated with implementation outcomes (fidelity)

Using previous papers as a model [15, 28], we conducted regression models to test whether each CFIR construct (independent variable) was associated with the 12-month implementation composite scores (PA and HE; dependent variables) after controlling for baseline practices. Analyses were limited to churches that met in person at baseline (*n* = 69) due to full availability of implementation outcomes for both PA and HE in this sample. A standardized regression coefficient (*β*) was computed for each model; effect sizes of *β* = |0.10| were considered small, *β* = |0.30| medium, and *β* = |0.50| large [27].

## Results

### Church and committee member characteristics

As reported previously [17], 107 churches from 23 US states representing 21 different denominations enrolled in the study. Two churches in the sample were served by the same pastor, and in four churches, the pastor served as the FAN coordinator (they were classified as FAN coordinators in all analyses). From the total sample, 69 churches were meeting only/mainly in person at baseline, and 38 only/mainly online. Ninety FAN coordinators (84.1%) completed the 12-month survey, and completion rates were comparable for churches meeting in-person (84.1%) versus online (84.2%). Characteristics of the total sample, and by meeting format and 12-month completion are shown in Table 2.

**Table 2:** Characteristics of churches (*N* = 107) and FAN coordinators in the Faith, Activity, and Nutrition (FAN) national implementation study, overall and by meeting format and by whether the FAN coordinator completed the 12-month survey

Characteristic	Full sample (*N* = 107)	Only/mainly in-person (*n* = 69)	Only/mainly online (*n* = 38)	Completed 12-month survey (*n* = 90)
Region of county, % (*n*)				
Southeast	75.7 (81)	76.8 (53)	73.7 (28)	76.7 (69)
Northeast	10.3 (11)	11.6 (8)	7.9 (3)	10.0 (9)
Midwest	6.5 (7)	4.4 (3)	10.5 (4)	7.8 (7)
Southwest	3.7 (4)	4.4 (3)	2.6 (1)	4.4 (4)
West	3.7 (4)	2.9 (2)	5.3 (2)	1.1 (1)
Predominant (≥80%) race/ethnicity of congregation, % (*n*)				
African American/Black	74.8 (80)	69.6 (48)	84.2 (32)	73.3 (66)
White	20.6 (22)	24.6 (17)	13.2 (5)	23.3 (21)
Other or Multiracial	4.6 (5)	5.8 (4)	2.6 (1)	3.3 (3)
Denomination, % (*n*)				
Methodist tradition	48.6 (52)	46.4 (32)	52.6 (20)	50.0 (45)
Baptist tradition	23.4 (25)	24.6 (17)	21.0 (8)	24.4 (22)
Nondenominational Christian	6.5 (7)	5.8 (4)	7.9 (3)	3.3 (3)
Pentecostal/Holiness tradition	8.4 (9)	10.1 (7)	5.3 (2)	8.9 (8)
Presbyterian	3.7 (4)	2.9 (2)	5.3 (2)	4.4 (4)
Lutheran	4.7 (5)	5.8 (4)	2.6 (1)	5.6 (5)
Other denomination	4.7 (5)	4.4 (3)	5.3 (2)	3.3 (3)
Worshipers per week, % (*n*)				
1–19	9.4 (10)	13.0 (9)	2.6 (1)	8.9 (8)
20–99	57.9 (62)	56.5 (39)	60.5 (23)	57.8 (52)
100+	32.7 (35)	30.4 (21)	36.8 (14)	33.3 (30)
Active health ministry, % (*n*)	51.4 (55)	50.7 (35)	52.6 (20)	48.9 (44)
Church had a pastor change, % (*n*)	11.2 (12)	8.7 (6)	15.8 (6)	13.3 (12)
FAN coordinator female, % (*n*) FAN coordinator education, % (*n*)	94.4 (101)	92.8 (64)	97.4 (37)	95.6 (86)
High school graduate or some college	27.1 (29)	29.0 (20)	23.7 (9)	27.8 (25)
College graduate	72.9 (78)	71.0 (49)	76.3 (29)	72.2 (65)
FAN coordinator age, mean (SD)	58.8 (11.7)	58.1 (11.1)	58.6 (13.0)	59.5 (12.0)

### Implementation outcomes (fidelity)

#### PA components

As shown in Table 3, there were significant Time × Meeting format interactions for the PA implementation composite, as well as for PA messages and PA policies, with greater increases seen for churches meeting only/mainly in person versus online at baseline. For the components of PA opportunities and PA pastor support, there were significant increases over time, but the Time × Meeting format interactions did not reach statistical significance. In terms of within-group effects, churches meeting only/mainly in person had a significant increase in the implementation of all components from baseline to 12 months, with effect sizes ranging from *d* = 0.59 to 1.18 (i.e. medium to large). Churches meeting only/mainly online had significant increases in all components except for PA policies and PA pastor support, with effect sizes ranging from *d* = 0.30 to 1.32 (i.e. small to large).

**Table 3:** Changes in implementation outcomes (fidelity) from baseline to 12 months, by meeting format at baseline

	Only/mainly in person (*n* = 69)			Only/mainly online (*n* = 38)				
Implementation outcome	Baseline LSM (SE)	12-month LSM (SE)	*d*	Baseline LSM (SE)	12-month LSM (SE)	*d*	Time *P*	Time × Meeting *P*
Physical activity composite	1.69 (0.08)	2.38 (0.09)	^*^1.12	1.49 (0.10)	1.89 (0.11)	^*^0.88	< .0001	.04
Opportunities	1.73 (0.08)	2.58 (0.09)	^*^1.16	1.39 (0.11)	1.98 (0.12)	^*^1.32	< .0001	.12
Messages	1.52 (0.09)	2.30 (0.10)	^*^1.04	1.42 (0.13)	1.80 (0.13)	^*^0.49	< .0001	.02
Policies	1.42 (0.10)	2.07 (0.10)	^*^1.18	1.26 (0.13)	1.48 (0.14)	0.35	< .0001	.01
Pastor support	1.92 (0.11)	2.42 (0.12)	^*^0.59	1.91 (0.15)	2.18 (0.16)	0.30	.001	.31
Healthy eating composite	2.01 (0.08)	2.69 (0.08)	^*^1.20	–	–	–	< .0001	–
Opportunities	2.89 (0.08)	3.35 (0.08)	^*^0.89	–	–	–	< .0001	–
Messages	1.58 (0.09)	2.34 (0.10)	^*^1.03	1.54 (0.13)	1.89 (0.14)	^*^0.45	< .0001	.046
Policies	1.56 (0.11)	2.39 (0.12)	^*^0.99	1.50 (0.15)	1.72 (0.17)	0.28	< .0001	.006
Pastor support	2.24 (0.10)	2.70 (0.11)	^*^0.56	2.30 (0.14)	2.41 (0.15)	0.13	.005	.08

*Note*: LSM = least square mean. SE = standard error. *d* = Cohen’s d (12-month LSM—baseline LSM)/baseline standard deviation.

^*^Indicates that the within-group change over time was statistically significant, *P* < .05. For HE opportunities, analyses were limited to churches that were meeting mainly/only in-person at baseline.

#### HE components

As shown in Table 3, there were significant Time x Meeting format interactions for HE messages and HE policies, with greater increases seen for churches meeting only/mainly in person versus online at baseline. For the component of HE pastor support, there was a significant increase over time, but the Time x Meeting format interaction did not reach statistical significance. There was a significant increase over time for HE opportunities for churches meeting only/mainly online (not analyzed for churches meeting online), and these increases were seen for each of the subitems: fruits, vegetables, whole grains, and healthy meats (data not shown). In terms of within-group effects, churches meeting mainly/only in person had a significant increase in the implementation of all HE components from baseline to 12 months, with effect sizes ranging from *d* = 0.56 to 1.20 (i.e. medium to large). Churches meeting only/mainly online significantly increased their implementation of HE messages but not HE policies or HE pastor support, with effect sizes ranging from *d* = 0.13 to 0.45 (small).

### CFIR constructs associated with implementation outcomes (fidelity)

#### Physical activity

As shown in Table 4, among churches meeting only/mainly in person at baseline and for which the FAN coordinator completed a baseline and 12-month survey (*n* = 58), CFIR constructs from each of the assessed domains were related to PA implementation. For the *intervention characteristics domain*, higher ratings of adaptability and lower ratings of complexity at 12 months were significantly associated with greater 12-month PA implementation. For the *inner setting domain*, lower tension for change (baseline), as well as greater compatibility (12 months), relative priority (12 months), organizational incentives/rewards (12 months), readiness for implementation (12 months), and congregant needs (baseline and 12 months) were significantly associated with greater 12-month PA implementation. For the *characteristics of the individual domain*, greater self-efficacy (baseline and 12 months) and perceived benefits (12 months) were significantly associated with greater 12-month PA implementation. Finally, for the *implementation process domain*, greater levels of engaging opinion leaders and engaging champions (12 months) were significantly associated with greater 12-month PA implementation.

**Table 4:** CFIR item means and associations with 12-month implementation composite scores for churches meeting only/mainly in-person (*n* = 58 churches)

		Physical activity		Healthy eating	
CFIR DOMAIN, Construct, and Item	Time	Mean (SD)	^a^Model β	Mean (SD)	^a^Model β
INTERVENTION CHARACTERISTICS					
**Adaptability**					
Can be adapted to fit church	T	4.06 (0.56)	0.16	4.31 (0.47)	0.10
	12M	3.23 (0.54)	0.34^**^	3.37 (0.72)	0.10
**Complexity**					
Easy to use	T	4.04 (0.64)	0.06	4.26 (0.68)	0.02
	12M	3.18 (0.55)	0.36^**^	3.40 (0.68)	−0.09
**Cost**					
Expensive (reverse scored)	BL	2.69 (0.61)	−0.16	2.38 (0.65)	−0.33^**^
	T	3.52 (1.09)	−0.10	3.52 (0.98)	−0.00
	12M	2.93 (0.66)	−0.08	2.84 (0.76)	0.24^*^
Great deal of time (reverse scored)	BL	2.68 (0.56)	0.18	2.81 (0.46)	0.06
	T	3.35 (1.03)	0.14	3.26 (1.03)	−0.02
	12M	2.67 (0.64)	0.03	2.79 (0.78)	0.26^*^
**Relative advantage**					
More effective than other programs	12M	3.02 (0.74)	0.24	3.02 (0.74)	0.18
**INNER SETTING**					
**Structural characteristics**					
Presence of health ministry	BL	0.51 (0.50)	0.00	0.51 (0.50)	0.12
Tenure of pastor	BL	2.80 (1.37)	0.04	2.80 (1.37)	0.03
Weekly attendance >100	IF	0.30 (0.46)	−0.09	0.30 (0.46)	−0.19
Predominantly African American	IF	0.70 (0.46)	0.12	0.70 (0.46)	0.08
Pastor change in past year	N/A	0.09 (0.28)	−0.20	0.09 (0.28)	−0.19
**Culture** ^b^	BL	3.45 (0.75)	−0.19	3.45 (0.75)	−.07
**Networks & Communication**					
Composite^c^	BL	3.45 (0.46)	0.17	3.45 (0.46)	0.15
Leaders involve members in decisions	BL	3.26 (0.64)	0.11	3.26 (0.64)	0.08
**Implementation Climate**					
** Tension for change**					
New ideas readily accepted	BL	3.18 (0.65)	−0.32^**^	3.18 (0.65)	−0.26^*^
Leaders like traditional ways (reverse scored)	BL	2.29 (0.78)	0.08	2.29 (0.78)	0.08
** Compatibility**					
Matches church priorities	BL	3.19 (0.72)	0.04	3.19 (0.72)	−0.04
	T	4.07 (0.77)	0.16	4.07 (0.77)	0.18
	12M	3.07 (0.62)	0.29^*^	3.07 (0.62)	0.42^***^
Fits with way you work	12M	3.26 (0.52)	0.30^*^	3.26 (0.52)	0.30^*^
** Relative priority**					
Health ministry as important as spiritual ministry	BL	3.27 (0.75)	−0.05	3.27 (0.75)	0.03
	12M	3.36 (0.67)	0.29^*^	3.36 (0.67)	0.35^**^
** Organizational incentives/rewards**					
Recognized for implementation	12M	3.15 (0.63)	0.41^***^	3.13 (0.72)	0.18
** Readiness for implementation**					
Received enough training	12M	3.27 (0.62)	0.14	3.45 (0.66)	0.11
Pastor encouraged congregants to embrace	12M	3.08 (0.76)	0.39^**^	3.13 (0.75)	0.42^***^
** Congregant needs**					
Well-received by most congregants	BL	3.07 (0.59)	0.26^*^	3.24 (0.56)	−0.06
	T	3.83 (0.69)	0.11	3.89 (0.74)	0.18
	12M	2.92 (0.76)	0.42^***^	3.02 (0.75)	0.31^*^
CHARACTERISTICS OF THE INDIVIDUAL (FAN Coordinator)					
**Beliefs about the intervention**					
Valuable for church	BL	3.43 (0.67)	0.20	3.56 (0.58)	0.05
	T	4.39 (0.71)	−0.01	4.61 (0.49)	−0.12
	12M	3.36 (0.62)	0.04	3.42 (0.63)	0.12
**Self-efficacy**					
Have skills to make changes	BL	3.03 (0.77)	0.20	3.23 (0.60)	0.06
	T	4.15 (0.63)	0.19	4.28 (0.66)	0.14
	12M	3.27 (0.56)	0.27^*^	3.38 (0.62)	0.05
Confident can make changes	BL	3.16 (0.64)	0.31^**^	3.35 (0.59)	0.06
	T	4.13 (0.64)	0.22	4.26 (0.62)	0.32^**^
	12M	3.17 (0.55)	0.47^***^	3.26 (0.65)	0.22
**Perceived benefits**					
Church will benefit (has benefited)	BL	3.49 (0.66)	0.22	3.67 (0.47)	0.02
	T	4.61 (0.49)	−0.02	4.61 (0.49)	0.01
	12M	3.08 (0.62)	0.47^***^	3.24 (0.77)	0.18
**Individual identification with organization** ^d^	BL	3.75 (0.52)	0.04	3.75 (0.52)	0.08
**Other personal attributes**					
Church membership >20 years	BL	0.58 (0.50)	−0.06	0.58 (0.50)	−0.19
Led health promotion efforts	BL	0.61 (0.49)	−0.01	0.61 (0.49)	0.04
Age, years	BL	58.81 (11.08)	−0.22	58.81 (11.08)	−0.14
Some college	BL	0.71 (0.46)	0.17	0.71 (0.46)	0.15
Women	BL	1.93 (0.26)	−0.04	1.93 (0.26)	−0.03
Meets public health recommendations for target behavior (PA, HE)	BL	0.54 (0.50)	0.01	0.28 (0.45)	−0.03
	12M	0.45 (0.50)	0.07	0.38 (0.49)	0.27^*^
IMPLEMENTATION PROCESS					
**Engaging opinion leaders**					
Leaders actively involved	12M	2.79 (0.70)	0.49^***^	2.92 (0.72)	0.38^**^
**Engaging champions**					
At least one person is champion	12M	3.38 (0.62)	0.34^**^	3.46 (0.63)	0.08

For time, IF = reported on interest form prior to enrollment, BL = baseline (pretraining), T = training (completed at the end of lesson 7), 12M = 12 months.

^*^ *P* < .05.

^**^ *P* < .01.

^***^ *P* < .001.

^a^β represents the standardized regression coefficient for each CFIR item predicting 12-month PA and HE implementation, adjusted for baseline practices.

^b^Culture score was an average of 2 items (*α* = 0.92): pastor has a sense of personal responsibility for improving congregant health, pastor is open to changes in church guidelines or policies that impact congregants.

^c^Composite score for networks and communication was an average of 3 items (*α* = 0.81): pastor and church leaders share information and knowledge, pastor has good working relationships with other church leaders, and there is very little tension or conflict between church members.

^d^Individual identification with organization score was an average of 2 items (*α* = 0.83): I want to perform to the best of my ability, I feel a strong sense of commitment to my church.

#### Healthy eating

As shown in Table 4, CFIR constructs from each of the assessed domains were also related to HE implementation. For the *intervention characteristics domain*, greater perceived cost (financial) at baseline but lower perceived cost (financial and time) at 12 months were significantly associated with greater 12-month HE implementation. For the *inner setting domain*, lower tension for change (baseline), as well as greater compatibility (12 months), relative priority (12 months), readiness for implementation (12 months), and congregant needs (12 months) were significantly associated with greater 12-month HE implementation. For the *characteristics of the individual domain*, greater self-efficacy (at end of training) and meeting HE guidelines for fruit and vegetable intake (12 months) were significantly associated with greater 12-month HE implementation. Finally, for the *implementation process domain*, a greater level of engaging opinion leaders (12 months) was significantly associated with greater 12-month HE implementation.

## Discussion

This national implementation study demonstrated that an online training format resulted in significant increases in the implementation of FAN components, particularly in churches where the FAN coordinator attended services and activities only/mostly in person (our proxy measure of how the church was operating). Further, the magnitude of these increases was medium to large. This study, along with our findings related to recruitment, enrollment, adoption, and training [17, 18], indicate that church committees are receptive to carefully designed online training and that the training results in positive organizational-level outcomes. Population reach and public health impact are greater in interventions that target organizational- versus individual-level change [25]. Our findings are noteworthy given challenges in this setting, including the lack of mandates for health promotion, the fact that improving physical health is not the primary mission of the church, the reliance on volunteers, and the lack of financial resources for health promotion [3]. They also highlight the potential to scale up an intervention using an online training format [29]. The majority of churches in our study represented predominantly African American congregations, and positive changes in this setting could enhance health equity. Despite a decline in religious affiliation since the 1990s, attendance at religious services remains high among Black adults in the USA [5].

Our team has conducted three prior studies of FAN, where the FAN coordinator, congregants, or both were surveyed regarding implementation [12, 14, 15, 30]. In each study, churches took part in a full-day, in-person training. Effect sizes observed in the current study were within the range of effect sizes observed in these prior studies, including our original group randomized trial [12], for the components of PA and HE opportunities, messages, and policies. Our findings suggest that FAN maintains its effectiveness despite changes in training modality. The effect sizes for both PA and HE pastor support, although of medium magnitude, were somewhat smaller than in previous studies, possibly because the completion rate of pastor training was low in this study (pastor training was optional in all studies). Although the FAN training emphasizes how the committee can enlist support from the pastor, as well as ways to facilitate that support, the pastor may need direct exposure to these messages to effectively support FAN. Given the many competing demands that pastors face, it may be more challenging for a pastor to find time each week over eight weeks to complete the training than to set aside a day for training.

This study also identified constructs from the CFIR related to PA and HE implementation. We used a similar approach to examining these associations as we did in a prior statewide initiative [15]. In both studies, we identified factors within the CFIR domains of intervention characteristics, inner setting, characteristics of the individual, and implementation process that related to 12-month PA and HE implementation. The constructs associated with PA and HE implementation differed by domain, with no overlap in associations in the intervention characteristics domain and substantial overlap in the inner setting domain. This finding is consistent with the observation that many churches traditionally serve food, and FAN focuses on incremental increases in healthier options. In contrast, PA is new to many churches, so they may not have the capacity and history for PA. Constructs shown to be important in both studies for the inner setting domain included tension for change (new ideas are readily accepted), relative priority (health ministry is as important as spiritual ministry), organizational incentives and rewards (recognized for implementation), readiness for implementation (pastor encouraged congregant to embrace FAN components), and congregant needs (well-received). Unlike our previous study [15], the item “new ideas are readily accepted” was associated with implementation outcomes in an unexpected direction, for which we have no explanation. Within the characteristics of the individual domain, both studies found that the FAN coordinator’s self-efficacy to make changes was associated with increased implementation. Finally, for the implementation process domain, engaging opinion leaders and engaging champions were significantly related to greater implementation in both studies. In our prior study, only relative advantage from the intervention characteristics domain was related to 12-month implementation. In contrast, the current study found adaptability, complexity, and cost to be significant correlates, but not relative advantage. A contradictory finding, where greater perceived cost at baseline and lower perceived cost at 12 months were associated with greater 12-month HE implementation, was not anticipated. Baseline costs were rated prior to FAN exposure, whereas 12-month costs were rated based on experience. It is possible that churches inclined to implement at baseline (for any number of reasons) perceived that it would be costly and the experience of implementing HE in FAN did not bear this out. Replication of important constructs can be useful for understanding characteristics of churches for which FAN may not be a good fit (e.g. the belief that health should be a focus of the church) as well as characteristics that can be enhanced via training and technical assistance (e.g. implementation recognition, engaging leaders, and champions).

Although studies in recent years have focused on faith-based interventions with an implementation science lens, many of these studies enrolled a relatively small number of churches or were limited to a single geographic area (e.g. [9, 31]), and most focused on individual-level (versus organizational-level) change. Furthermore, few studies have applied implementation science frameworks such as the CFIR to better understand factors that influence implementation outcomes, and when they have, the studies have often been qualitative [10, 32, 33]. There are exceptions. For example, CRUZA was an organizational intervention conducted with 65 Roman Catholic parishes in Massachusetts [34] that used quantitative items to measure CFIR constructs to predict implementation of evidence-based interventions for cancer control. They found few differences between parishes that implemented and those that did not. The size and scope (US) of our study, its focus on organizational change, and the quantitative examination of CFIR constructs associated with implementation make substantial contributions to faith-based implementation research.

Several limitations of this study should be considered. First, we used a quasi-experimental design without a comparison group (see [20]). Because our primary study focus is on implementation outcomes and factors that influence them rather than comparing different implementation strategies, randomization is an unnecessary cost and burden. This approach is consistent with the call for more pragmatic trials [35] and for approaches that study rather than attempt to minimize contextual factors [36]. Further, randomization is not favorably viewed by our church partners. Second, despite our attempts to reach churches across the entire USA, we had an overrepresentation from the Southeast and enrolled churches in less than half of US states. Nonetheless, religiosity and church attendance are greater in the Southeast than in other regions of the country, and the prevalence of chronic disease is higher, underscoring the need and fit. Third, we relied on self-reported implementation. It would not have been feasible to conduct site visits and independent ratings given the national focus of the study. It is noteworthy that in past studies, effect sizes were similar when reported by the FAN coordinator versus congregants [12, 14, 15, 30]. Fourth, despite recruiting perhaps the largest sample of churches to date for a PA and HE intervention, our sample was still relatively small for examining constructs associated with implementation. Further, our CFIR analyses were limited to churches meeting in-person at baseline, further reducing our sample size. As a result, we did not control for type I error and instead included the effect sizes so that readers can understand the magnitude of effects. Future research might benefit from employing path models or structural equation modeling to investigate more complex interactions.

Despite these limitations, this study contributes to the call for more implementation research in faith-based settings [3, 6]. We have demonstrated consistency in implementation outcomes for FAN when trained via study staff (in person), community health advisors (in person), and an online format. The online format has great potential for wider dissemination and reach. We have also created a model for mapping quantitative items onto CFIR constructs and related them to implementation outcomes. This approach could be used in a variety of community settings to better understand how to maximize implementation.

## Supplementary Material

ibaf015_suppl_Supplementary_Files_1

ibaf015_suppl_Supplementary_Files_2

## Welfare of Animals

This article does not contain any studies with animals performed by any of the authors.

## Transparency Statements

(1) Study registration: This study was not formally registered; (2) Analytic Plan Pre-Registration: The analysis plan was not formally preregistered; (3) Data Availability: De-identified data from this study are not available in a public archive. De-identified data from this study will be made available upon reasonable request and as allowable according to institutional IRB standards by emailing the corresponding author; (4) Analytic Code Availability: Analytic code used to conduct the analyses presented in this study are not available in a public archive. They may be available by emailing the corresponding author; (5) Materials Availability: Materials used to conduct the study are not publicly available. They may be available by emailing the corresponding author.
