# Supplementary material for: Implementation outcomes and associated constructs from the Consolidated Framework for Implementation Research among churches trained online to implement Faith, Activity, and Nutrition in a national implementation study
Source: Transl Behav Med. 2025 May 29;15(1):ibaf015. doi: 10.1093/tbm/ibaf015 (PMC12169341; doi:10.1093/tbm/ibaf015)
Supplement: ibaf015_suppl_Supplementary_Files_1 [file ibaf015_suppl_supplementary_files_1.docx]

Supplemental File 1. FAN Implementation Outcome Measures of Fidelity

| **Variable** | **Items** | **Response options** | **Questions** |
| --- | --- | --- | --- |
| Physical activity opportunities – incorporated into church activities | 2 | 1=Not at all  2=Very little  3=Some of the time  4=Almost all of the time | Currently, how often are opportunities to be physically active included before, during or after worship service (including online services)?  Currently, how often are opportunities to be physically active included in existing meetings and events (including online meetings and events)? |
| Physical activity opportunities – programs | 2 | 1=Rarely or never  2=Every few months  3=About monthly  4=About weekly | Currently, how often are physical activity programs offered at your church (e.g., walking programs, sports leagues, exercise classes)?  Currently, how often are church members made aware of free or low-cost physical activity opportunities in the community? |
| Physical activity messages | 3 | 1=Rarely or never  2=Every few months  3=About monthly  4=About weekly | Currently, how often is new information about physical activity posted on a bulletin board (or other visible location) in your church?  Currently, how often do members receive church bulletins, bulletin inserts, or handouts that include messages about physical activity (printed or electronic)?  Currently, how often does someone, other than the pastor, share messages about physical activity during worship services? |
| Physical activity policies | 1 | 1=No, not at this time  2=No, but it's now being considered  3=Yes, it's partially in place  4=Yes, it's fully in place | Currently, is a church guideline or policy in place that states physical activity will be included in church meetings and events? |
| Physical activity pastor support | 3 | 1=Rarely or never  2=Every few months  3=About monthly  4=About weekly | Currently, how often does the pastor include messages about physical activity during worship services? (including online services)  Currently, how often does the pastor make positive or supportive comments about physical activity? (consider in-person, online, print, and electronic forms of communication)  Currently, how often does the pastor take part in physical activity? |
| Healthy eating opportunities | 4 | 1=Not at all  2=Very little  3=Some of the time  4=Almost all of the time | Currently, how often are (*item below*) available to church members at church functions that include food?   - fruits or fruit dishes (with no or very little sugar added) - vegetables or vegetable dishes (low in fat and sodium) - meats that were not fried and without skin - whole grains (e.g., brown rice, whole wheat bread, whole wheat pasta) |
| Healthy eating messages | 3 | 1=Rarely or never  2=Every few months  3=About monthly  4=About weekly | Currently, how often do members receive church bulletins, bulletin inserts, or handouts that included messages about healthy eating (printed or electronic)?  Currently, how often does someone, other than the pastor, share messages about healthy eating during worship services? (including online services)  Currently, how often is new information about healthy eating posted on a bulletin board (or other visible location) in your church? |
| Healthy eating policies | 1 | 1=No, not at this time  2=No, but it's now being considered  3=Yes, it's partially in place  4=Yes, it's fully in place | Currently, is a church guideline or policy in place that states healthy food choices (fruits, vegetables, whole grains, low fat, and/or low sodium) will be included in all meetings and events when food is served? |
| Healthy eating pastor support | 3 | 1=Not at all  2=Very little  3=Some of the time  4=Almost all of the time  1=Rarely or never  2=Every few months  3=About monthly  4=About weekly | Currently, when the pastor attends church functions that include food, how often does the pastor choose healthy food options?  Currently, how often does the pastor include messages about healthy eating during worship services? (including online services)  Currently, how often does the pastor make positive or supportive comments about healthy eating? (consider in-person, online, print, and electronic forms of communication) |
