# Supplementary material for: Implementation outcomes and associated constructs from the Consolidated Framework for Implementation Research among churches trained online to implement Faith, Activity, and Nutrition in a national implementation study
Source: Transl Behav Med. 2025 May 29;15(1):ibaf015. doi: 10.1093/tbm/ibaf015 (PMC12169341; doi:10.1093/tbm/ibaf015)
Supplement: ibaf015_suppl_Supplementary_Files_2 [file ibaf015_suppl_supplementary_files_2.docx]

Supplemental File 2. Domains, Constructs, and Items from the Consolidated Framework for Implementation Research (CFIR) [[1](#_ENREF_1)] Measured in the Faith, Activity, and Nutrition (FAN) National Implementation Study

| **Construct** | **Description** | **Items & source** | **Time of administration** |
| --- | --- | --- | --- |
| **Domain 1: Intervention/Program Characteristics** | | | |
| Adaptability | The degree to which an intervention can be adapted, tailored, refined, or reinvented to meet local meets | The [healthy eating / physical activity] parts of FAN can be adapted to fit your church. [[2](#_ENREF_2)] | T, 12M |
| Complexity | Perceived difficulty of implementation based on duration, scope, disruptiveness, centrality, steps to implement | If your church chose to provide [healthy food choices / opportunities for physical activity], it would be easy to do so. [[2](#_ENREF_2)]  The [healthy eating / physical activity] parts of FAN are easy to use. [[2](#_ENREF_2)] | T  12M |
| Cost | Costs associated with implementing the intervention, including investment, supply, and opportunity costs | If your church chose to provide [healthy food choices / opportunities for physical activity], it would be expensive to do so.  If your church chose to provide [healthy food choices / opportunities for physical activity], it would take a great deal of time to do so.  The [healthy eating / physical activity] parts of FAN are expensive to put in place.  The [healthy eating / physical activity] parts of FAN take a great deal of time to put in place. | BL  T, 12M |
| Relative advantage | The perceived advantage of implementing the intervention versus an alternative program | FAN is more effective than other health programs your church has used. [[2](#_ENREF_2), [3](#_ENREF_3)] | 12M |
| **Domain 3: Inner Setting** | | | |
| Structural characteristics | The social architecture, age, maturity, and size of an organization | Presence of health ministry - Does your church have a health ministry? A health ministry is a recognized team of people who sponsor regular educational events and experiences that promote well-being. (Yes, No)  Tenure of pastor – How long has your pastor / church leader been the leader at your church? (less than 1 year, 1-5 years, 6-10 years, 11-20 years, more than 20 years)  Weekly worship attendance – On average, about how many people attend worship service(s) each week at your church? (1-19, 20-99, 100-499, 500 or more)  Predominant race of congregation - Please estimate what percentage (%) of worshipers in your church belong to each of the following race groups. Please enter a whole number between 0 and 100 only. (White, Black or African American, American Indian or Alaska native, Asian, Native Hawaiian or Other Pacific Islander, Some other race) (also asked of pastor at baseline) [[4](#_ENREF_4)]  Please estimate what percentage (%) of worshipers in your church are of Hispanic, Latino, or Spanish origin. Please enter a whole number between 0 and 100 only.(also asked of pastor at baseline) [[4](#_ENREF_4)]  Pastor change in past year (Yes, No) | BL  IF  Documented by staff |
| Culture | Norms, values, and basic assumptions of a given organization | Your pastor has a sense of personal responsibility for improving congregant health. [[5](#_ENREF_5)]  Your pastor is open to changes in church guidelines or policies that impact congregants. [[6](#_ENREF_6)] | BL |
| Networks & communications | The nature and quality of webs of social networks and nature and quality of formal and informal communications within an organization | Your pastor and church leaders actively share information and knowledge with each other. [[2](#_ENREF_2)]  Your pastor has good working relationships with other church leaders. [[2](#_ENREF_2)]  There is very little tension and conflict between members in your church. [[6](#_ENREF_6)]  Leaders in your church involve members when decisions are made. [[6](#_ENREF_6)] | BL |
| Implementation climate   - Tension for change | The capacity for change, shared receptivity to an intervention, and the extent to which use of the intervention will be rewarded, supported, and expected within the organization | Tension for change  New ideas are readily accepted in your church. [[6](#_ENREF_6)]  Leaders in your church like to keep to established, traditional ways of doing things. [[6](#_ENREF_6)] | BL |
| - Compatibility |  | Compatibility  Providing healthy food choices for meals and snacks and providing opportunities for physical activity matches the priorities of your church. [[7](#_ENREF_7)]  FAN matches the priorities of our church. [[7](#_ENREF_7)]  Using FAN fits well with the way I like to work. [[2](#_ENREF_2)] | BL  T, 12M  12M |
| - Relative priority |  | Relative priority  The health ministry is as important as the spiritual ministry in your church. | BL, 12M |
| - Organizational incentives & rewards |  | Organizational incentives & rewards  You are recognized in your church for carrying out the [healthy eating / physical activity] parts of FAN. [[8](#_ENREF_8)] | 12M |
| Readiness for implementation   - Available resources | Tangible and immediate indicators of organizational commitment to its decision to implement and intervention | Available resources:  You received enough training to carry out the [healthy eating / physical activity] parts of FAN in your church. [[5](#_ENREF_5), [6](#_ENREF_6)] | 12M |
| - Leadership engagement |  | Leader engagement:  Your pastor encouraged congregants to embrace the [healthy eating / physical activity] parts of FAN. [[7](#_ENREF_7)] | 12M |
| Congregant needs and preferences | The extent to which the intervention takes into consideration congregant preferences and reactions | [Serving healthy food choices / providing opportunities for physical activity] would be well-received by members of your church. [[3](#_ENREF_3)]  I think the [healthy eating / physical activity] changes for FAN will be well-received by members of my church. [[3](#_ENREF_3)]  The [healthy eating / physical activity] parts of FAN have been well received by most of the congregants. [[3](#_ENREF_3)] | BL  T  12M |
| **Domain 4: Characteristics of Individuals involved with implementation (i.e., implementers)** | | | |
| Beliefs about the intervention | Attitudes toward and value placed on the intervention as well as familiarity with facts, truths, and principles related to the intervention | [Serving healthy food choices / providing opportunities for physical activity] would be valuable for your church.  The [healthy eating / physical activity] parts of FAN are valuable for your church. | BL  T, 12M |
| Self-efficacy | Individuals’ beliefs in their own capabilities to execute courses of action to achieve implementation goals | If your church chose to provide [healthier food choices opportunities for physical activity], you would have the skills needed to help your church do so. [[7](#_ENREF_7)]  If your church chose to provide [healthier food choices / opportunities for physical activity], you are confident that you could help your church do so. [[7](#_ENREF_7)]  You have the skills that are needed to make the [healthy eating / physical activity] changes for FAN work. [[7](#_ENREF_7)]  You are confident that you can make the [healthy eating / physical activity] changes for FAN. [[7](#_ENREF_7)]  You are confident that you will be able to continue to make the [healthy eating / physical activity] changes for FAN. [[7](#_ENREF_7)] | BL  T, 12M  T  12M |
| Perceived benefits | The phase an individual is in during progression toward skilled, enthusiastic, and sustained use of intervention | If your church chose to provide [healthier food choices / opportunities for physical activity], your church would benefit. [[7](#_ENREF_7)]  I think my church will benefit from the [healthy eating / physical activity] changes for FAN. [[7](#_ENREF_7)]  Your church has benefited from the [healthy eating / physical activity] changes made as part of FAN. [[7](#_ENREF_7)] | BL  T  12M |
| Individual identification with organization | How individuals perceive the organization and their relationship and commitment to it | You want to perform to the best of your ability for your church. [[6](#_ENREF_6), [8](#_ENREF_8)]  You feel a strong sense of commitment to your church. | BL |
| Other personal attributes | Other personal traits of the implementer | How long have you been a member of your church? (less than 1 year, 1 to 5 years, 6 to 10 years, 11 to 20 years, more than 20 years)  Have you led or co-led any health promotion efforts at your church or elsewhere in the past year? (Yes, No)  Age [[9](#_ENREF_9)] – What is your age (in years)?  Education [[9](#_ENREF_9)] – What is the highest grade or year of school you completed? (never attended school or only attended kindergarten, grades 1 through 8 – elementary, grades 9 through 11 – some high school, grade 12 or GED – high school graduate, college 1 to 3 years – some college or technical school, college 4 years or more – college graduate)  Gender identity [[9](#_ENREF_9)] – What is your gender identity? (male, female, other)  Meeting guidelines for fruit and vegetable intake [[10](#_ENREF_10)] - The next question asks you to report how many cups of fruit you eat each day.    One (1) cup of fruit is equal to:  • 1 cup of chopped fruit (fresh, canned, frozen)  • 1 cup of 100% fruit juice  • ½ cup of dried fruit  • 1 small apple  • 1 medium pear  • 1 large banana, peach, orange  • 2 large plums  • 8 large strawberries  About how many cups of fruit do you eat or drink each day?  The next question asks you to report how many cups of vegetables you eat each day.      One (1) cup of vegetables is equal to:  • 1 cup of raw or cooked vegetables  • 1 cup of 100% vegetable juice  • 2 cups of raw leafy greens (e.g., spinach, romaine)  • 3 5-inch spears of broccoli  • 12 baby carrots  • 2 large celery stalks  • 1 large bell pepper, raw tomato, or baked sweet potato  • 1 medium boiled or baked potato  • 1 cup of whole or mashed beans or peas (e.g., black,  garbanzo, kidney, pinto, black eye)  • 1 large ear of corn  About how many cups of vegetables do you eat or drink each day?  Meeting guidelines for physical activity [[9](#_ENREF_9)] – We are interested in two types of physical activity – moderate and vigorous.  Moderate activities cause small increases in breathing or heart rate while vigorous activities cause large increases in breathing or heart rate.  In a usual week (not including time at work), do you do moderate or vigorous activities such as brisk walking, bicycling, gardening, running, aerobics, heavy yard work, or anything else that causes small to large increases in breathing or heart rate? (Yes, No)  How many days per week do you do these moderate or vigorous intensity activities?  On days when you do moderate or vigorous activities, how much total time per day do you spend doing these activities? | BL  BL, 12M |
| **Domain 5: Implementation Process** | | | |
| Engaging   - Opinion leaders | Attracting and involving appropriate individuals in the implementation. Includes opinion leaders, champions, and external change agents | Opinion leaders:  Leaders in your church are actively involved in the [healthy eating activities for / physical activity parts of] FAN. [[2](#_ENREF_2)] | 12M |
| - Champions |  | Champions:  There is at least one person in your church who is a champion for the [healthy eating / physical activity] parts of FAN. [[3](#_ENREF_3)] | 12M |

FAN = Faith, Activity, and Nutrition. IF = reported on church interest form, BL = baseline (pre-training), T = training (completed at the end of lesson 7), 12M = 12 months. Unless otherwise noted, response options were: strongly disagree (1), disagree (2), agree (3), or strongly agree (4)

Data sources (CFIR items were adapted to reference the church, church leaders, and components of FAN):

1. Damschroder LJ, et al. Fostering implementation of health services research findings into practice: a consolidated framework for advancing implementation science*.* Implement Sci. 2009;4:50.

2. Cook JM, et al. Measurement of a model of implementation for health care: toward a testable theory*.* Implement Sci. 2012;7:59.

3. Thaker S, et al. Program characteristics and organizational factors affecting the implementation of a school-based indicated prevention program*.* Health Educ Res. 2008;23:238-48.

4. Polson EC and Dougherty KD. Worshiping across the color line: the influence of congregational composition on whites’ friendship networks and racial attitudes*.* Sociology of Race & Ethnicity. 2019;5:100-114.

5. Helfrich CD, et al. Organizational readiness to change assessment (ORCA): development of an instrument based on the Promoting Action on Research in Health Services (PARIHS) framework*.* Implement Sci. 2009;4:38.

6. Patterson MG, et al. Validating the organizational climate measure: links to managerial practices, productivity and innovation*.* J Organ Behav. 2005;26:379-408.

7. Holt DT, et al. Readiness for organizational change: the systematic development of a scale*.* J Appl Behav Sci. 2007;43:232-255.

8. Fernandez ME, et al. Developing measures to assess constructs from the Inner Setting domain of the Consolidated Framework for Implementation Research*.* Implement Sci. 2018;13:52.

9. Centers for Disease Control and Prevention. Behavioral Risk Factor Surveillance System Survey Questionnaires. 2009 [cited 2019 August 13]; Available from: <http://www.cdc.gov/brfss/questionnaires.htm>.

10. Resnicow K, et al. Body and soul. A dietary intervention conducted through African-American churches*.* Am J Prev Med. 2004;27:97-105.
